# Utilization of brewery wastes in food industry

**DOI:** 10.7717/peerj.9427

**Published:** 2020-07-14

**Authors:** Kamila Rachwał, Adam Waśko, Klaudia Gustaw, Magdalena Polak-Berecka

**Affiliations:** Department of Biotechnology, Microbiology and Human Nutrition, University of Life Sciences in Lublin, Lublin, Poland

**Keywords:** Brewery by-products, Waste re-use, Brewer’s spent grain-based food products, Food products from wastes

## Abstract

Beer is the most popular low-alcohol beverage consumed in large amounts in many countries each year. The brewing industry is an important global business with huge annual revenues. It is profitable and important for the economies of many countries around the world. The brewing process involves several steps, which lead to fermentation of sugars contained in malt and conversion thereof into alcohol and carbon dioxide by yeasts. Beer brewing generates substantial amounts of by-products. The three main brewing industry wastes include brewer’s spent grain, hot trub, and residual brewer’s yeast. Proper management of these wastes may bring economical benefits and help to protect the environment from pollution caused by their excessive accumulation. The disposal of these wastes is cumbersome for the producers, however they are suitable for reuse in the food industry. Given their composition, they can serve as a low-cost and highly nutritional source of feed and food additives. They also have a potential to be a cheap material for extraction of compounds valuable for the food industry and a component of media used in biotechnological processes aimed at production of compounds and enzymes relevant for the food industry.

## Introduction

**Figure 1:**
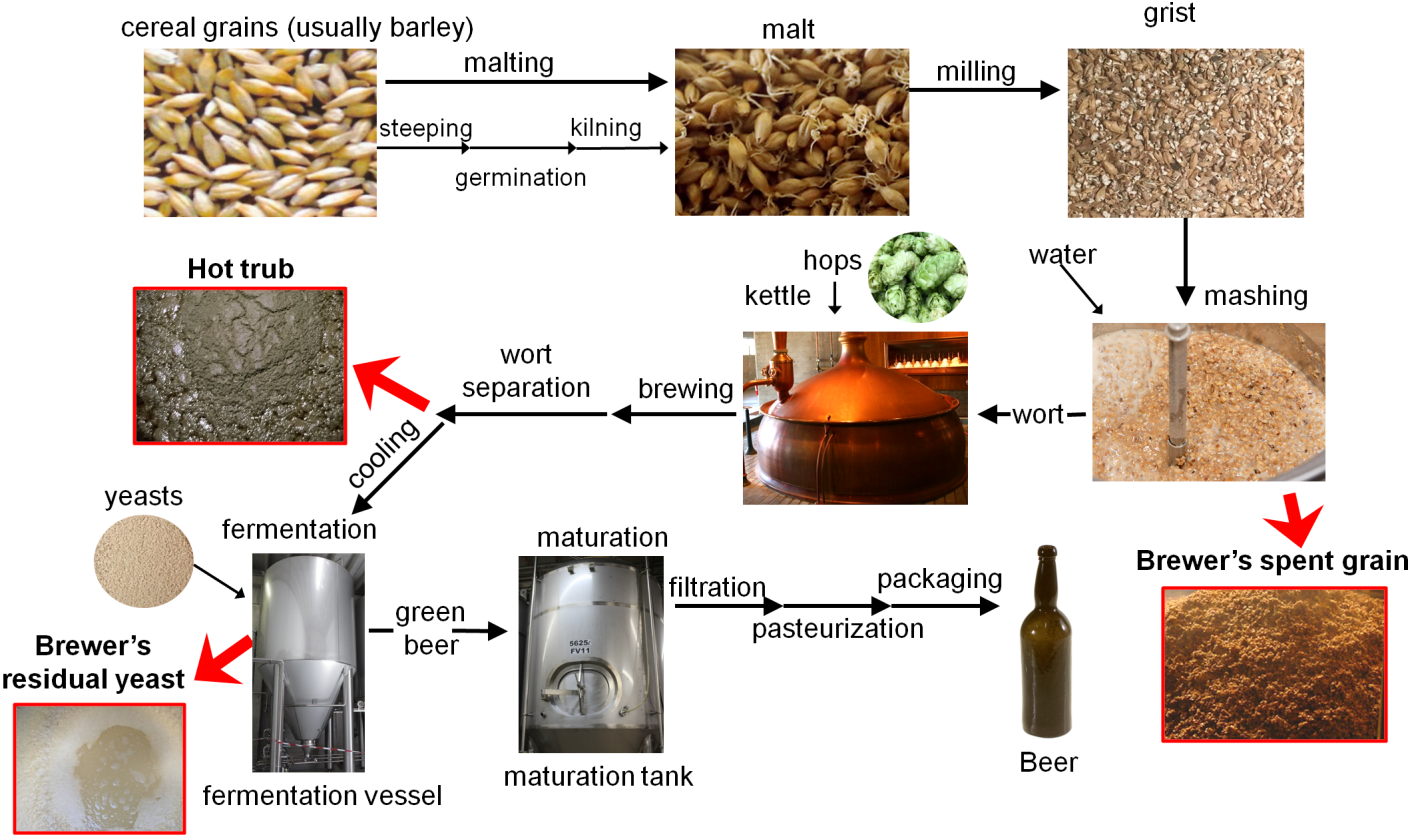
Beer production process (based on Mathias, de Mello & Servulo (2014)). Bigger red arrows indicates steps where the main brewery by-products are removed. Photo credit: CC search tool (Creative Commons, 2020).

Beer is a fermented beverage known since ancient times. Currently, it is the fifth most frequently consumed drink on a global scale. Global beer consumption in 2018, in 170 major countries and regions, was approximately 1.8879 billion hektoliters (Kirin Holdings Company, 2019). As reported by Conway (2019), global beer production exceeded 1.94 billion hL in 2018, which makes brewing one of the industries with significant economic importance. Modern brewing is mainly a large-scale industry producing substantial quantities of beer and by-products. Beer production is divided into several successive operations: malting, milling, mashing, lautering, adding hops or hop extract and boiling the beer wort with these additives, disposal of spent hops and precipitated trub, cooling the wort and aeration, fermentation with yeasts, removal of yeast, conditioning (maturation, aging) and packaging (Fig. 1). The aim of the process is to convert grain-derived starch into simple sugars, extract these sugars, and ferment them with the use of yeast to produce a lightly carbonated beverage with low alcohol content. Such a big-scale production results in generation of large quantities of organic waste materials by the brewing industry. The first and most abundant brewery by-product is formed after the mashing process. Residual “spent” grain is removed after the separation of the liquid produced during mashing (named wort). Another type of waste is generated after boiling the wort in the kettle. At this stage, thermal denaturation of the proteins takes place, which causes the precipitation of high molecular weight proteins (waste called hot trub). Hot trub, which contains spent hops, is then removed from the wort using a separator. Next, yeasts are added and the fermentation begins. After this step, most yeasts are removed (brewery spent yeast) from the young beer. Prior to packaging, beer is most often filtered through diatomaceous earth or cellulose filters to remove remaining yeast residues (Fig. 1) (Mathias, de Mello & Servulo, 2014; Young, 2019).

The brewery industry generates huge amounts of wastes, the management of which is economically troublesome. Their accumulation in the environment is an ecological issue as well. The increasing public concerns about environmental pollution have prompted the search for ways to reduce the production of industrial waste. The food industry is trying to find new applications that will change the traditional approach to ‘waste’ products and make them ‘co-products’ (Faˇrcaş et al., 2017). With their properties, by-products generated by the brewing industry have a potential to be applied as materials exploited in the food industry, but their use is still quite limited. Modern food science and technology aim to valorize food industry by-products for the production of chemicals, raw materials, and other value-added compounds (Helkar, Sahoo & Patil, 2016). Although studies focused on the reuse of brewery by-products are being conducted, there are no comprehensive review articles concerning all three types of brewery wastes showing its potential to be used in the food industry. This work is intended to summarize and disseminate research outcomes in this field. This will be particularly useful for brewers, food technologists, agricultural scientists, and some biotechnologist. The findings described have a practical nature through the possibility of being implemented. Knowledge of their potential may help to design new food and feed products from wastes or ways to recover functional components. The aim of this review was to summarize the possible utilization of the three main brewery wastes by the food industry and encourage search for new ways to exploit thereof.

### Survey methodology

At the initial stage of designing the review, the research question was formulated. To ensure that a review in this area is needed, the research area was scanned. The focus was placed on literature reviews and other available articles in this field. This step has resulted in a conclusion that the greatest contribution to the subject matter that we have undertaken would be made by a scoping review, which will summarize and disseminate research findings. Next, the target audience that might be interested in the article was considered. Additionally, a search strategy was developed to identify studies that are relevant to the selected topic. Based on words directly related to the topic of utilization of brewery wastes in the food industry, such search terms as brewery wastes, brewer’s spent grain, hot trub, residual brewer’s yeast, possibilities of reuse of brewery by-products (in food, feed, and production of food additives) and products made of brewery wastes were selected. References were collected from the selected databases, including PubMed, Web of Science, and Google Scholar. To access proper articles and books, inclusion and exclusion criteria were established. Literature was selected based on the year of publication (the latest articles were selected where possible), the language of the article (publications in languages other than English and Polish were excluded), and the significance of results (studies that did not address this research question were excluded). Next, the selected articles were analyzed using the same approach to abstract information relevant to the topic from each article. Subsequently, data from the literature was used to discuss the topic of this review.

## Brewing Industry Wastes

During beer production, three main types of by-products are generated, i.e. brewer’s spent grain, spent hops/hot trub, and residual brewer’s yeast (Fig. 1) (Mathias, de Mello & Servulo, 2014). They slightly differ between each other in some of the properties (composition, moisture, and ash content), but all of them can potentially be reused by the food industry. The chemical composition of brewery wastes can slightly vary depending on the type and quality of the ingredients used and the conditions prevailing during each step of the brewing process; nevertheless, they always have a high nutritional value. They are rich in carbohydrates, proteins, fibers, vitamins, minerals, and phenolic compounds and have high moisture content (since 1/5 of the water used in the brewing process is lost in the form of residues) (Santos et al., 2003; Olajire, 2012). This section outlines the general characteristics of the three main brewery wastes.

### Brewer’s spent grain (BSG)

Brewer’s spent grain, which accounts for approx. 85% of all residues produced by the brewing industry, is the major waste generated during beer production (Aliyu & Bala, 2011). BSG is formed in the mashing process and removed before the boiling step of the brewing process. This solid residue from wort production is composed of barley grain husks (Kerby & Vriesekoop, 2017). BSG is a heterogeneous material consisting of lignocellulosic biomass and is rich in proteins (20-30%), fiber (30–70%), lipids, vitamins, and minerals. It contains ca. 12–28% of lignin, 12–25% of cellulose, and 28% of non-cellulosic polysaccharides, mainly arabinoxylans (Mussatto & Roberto, 2005; Lynch, Steffen & Arendt, 2016). The chemical composition of BSG reported in the literature was previously determined and reviewed by Aliyu & Bala (2011). The amount of BSG produced is ca.14 kg/ hL wort, with moisture content between 75 % and 90% (Robertson et al., 2010). The ash content in brewer’s spent grain is in the range of 2.3–7.9 % (Aliyu & Bala, 2011). It has been evidenced that BSG contains vitamins, minerals, and many amino acids. This by-product is also rich in oligo- and polysaccharides and phenolic compounds (Cooray, Lee & Chen, 2017). Among phenolic acids, BSG has the highest content of ferulic (1860–1948 mg/g) and p-coumaric (565–794 mg g−1) acids as well as sinapic (Stefanello et al., 2018), caffeic, and syringic acids (Moreira et al., 2013).

### Hot trub

Another waste generated during manufacture of beer is hot trub. Hot trub is a term referring to sediments formed in the brewing process during wort boiling. The size of hot trub particles is estimated to be in the range of 30 to 80 µm (Kühbeck, Back & Krottenthaler, 2006; Mathias et al., 2015). This insoluble precipitate mainly consists of colloidal proteins coagulated during wort boiling, which form complexes with polyphenols that naturally occur in the wort. It also contains complex carbohydrates, lipids, minerals, tannins, hop residues, and smaller malt particles (Hough et al., 1982). In general, these residues, representing 0.2–0.4% of the wort volume, are removed as a by-product during the wort production process before fermentation. However, in some cases, hops can be added and removed at different stages of the brewing process. Approximately 85% of hops used for beer production are removed as a by-product (Huige, 2006).

Hot trub has high moisture content (between 80 % and 90%), approx. 15-20%dry matter content, and low ash content (approx. 2–5%) (Huige, 2006). It mainly consists of high-molecular-mass proteins; however, it has been reported to have a high concentration of carbon, which is a result of the high amount of reducing sugars (20%) in this residue (Mathias et al., 2015). The protein content in hot trub depends on several factors and may vary between breweries, although the average values range between 40 and 70% (Barchet, 1993; Kühbeck, Back & Krottenthaler, 2006). Hot trub exhibits a low C/N ratio (6.3).

The formation of trub is a highly desirable process. It is important to remove a considerable amount of the main trub components (polyphenols and soluble proteins) during the brewing process, as they can react and form insoluble complexes. They are visible as precipitates in beer, and are undesirable in pale filtered beers, which are expected to be bright and clear (Hough et al., 1982).

### Brewer’s spent yeast (BSY)

Brewer’s residual yeast is the second largest by-product from the brewing process (Huige, 2006). This slurry residue accounts for maximum 15% of total by-products generated during the brewing process (Kerby & Vriesekoop, 2017). BSY is recovered by sedimentation before full maturation of beer at the final stage of the second fermentation and maturation (Olajire, 2012). The excess yeast can be collected and re-used maximum six times. *Saccharomyces cerevisiae* yeast added at the beginning of fermentation during this process can undergo many divisions, which results in a considerably increased yeast biomass. The yeast growth rate depends on the fermentation conditions in the brewery. BSY generates beer losses in a range from 1.5 to 3% of the total volume of produced beer (Fillaudeau, Blanpain-Avet & Daufin, 2006). It was established that the average volume of residual yeast obtained from lager fermentation is 0.6–0.8 lb/bbl (2.7 kg/m^3^) of the final volume of beer (Huige, 2006). Yeasts are single-cell organisms containing proteins (49%), carbohydrates (40%), minerals and vitamins (7%), and lipids (4%). Brewer’s residual yeast exhibit high moisture content, i.e., approximately 74%–86%. Depending on the source, the mineral residue (ash) value for spent yeast is in the range of 2%–8.5% and its dry matter content is about 10–16% (Mathias et al., 2015). It has been reported that the mineral composition of *S. cerevisiae* may vary at different stages of the fermentation process. It also changes with subsequent reuses. Young yeast that has not been exploited many times is richer in phosphate and, hence, has greater ash content. BSY is also rich in polyphenolic compounds and B-group vitamins (mainly B1, B2, B3, B6, and B8) (Podpora et al., 2016). Analyses have revealed that brewer’s residual yeast exhibits high carbon content, with a value between 45% and 47% of yeast dry matter. The carbon/nitrogen ratio in this residue is 5.1–5.8 (Mathias et al., 2015).

## Applications of Brewery By-Products in the Food Industry

The brewery wastes discussed are used in various branches of the food industry, mainly as a feed additive and food ingredient. They can also serve as raw material for extraction of compounds used in the food industry or can be applied in biotechnological processes in which food industry additives are obtained.

### Brewery industry by-products as animal feed

Among the by-products of the brewing industry, BSG is most often sold as animal feed due to its properties and content of essential nitrogen-containing nutrients. It is used in a wet or dry final form as feed for livestock, poultry, pigs, goats, and fish (Dhiman, Bingham & Radloff, 2003; Mussato, 2006). Wet spent grains can serve as cake for ruminants, whereas dry SG is often used as feed for monogastric animals. The high moisture content makes it easily digestible to livestock (Olajire, 2012; Kerby & Vriesekoop, 2017). It has been demonstrated that sun-dried BSG can be included into pigs’ diet in a dose between 17% and 25% to improve the profitability of production (Amoah et al., 2017). Dry BSG also represents an alternative protein source for ruminants and can replace soybean. It is cheaper, but simultaneously has a comparable nutritional value (Faccenda et al., 2017). BSG can provide ruminants with all essential amino acids when administered with cheap nitrogen sources. It has been shown that up to 75% of soybean can be replaced by BSG in feed for lactating cows. In this amount, it increases digestibility and milk production (Faccenda et al., 2017). BSG was found to have a positive influence on the production efficiency in cattle, without affecting fertility. It improves the milk yield and composition and does not negatively influence blood components of dairy cows or dry matter intake (Belibasakis & Tsirgogianni, 1996; Chiou et al., 1998). Addition of BGS as feed for lactating cows increases the fat and protein content in milk (West, Ely & Martin, 1994; De Souza et al., 2016). BSG addition (at the level of 35%) to the lamb diet was found to exert a positive effect on their growth performance (higher body weight and daily gains). Moreover, it was shown to improve the quality of their meat. The meat exhibited lower fattiness and increased levels of linoleic cis- 9 and trans-11 PUFA acids. These results indicate that BSG can be used as low-cost feed for lambs, which additionally improves meat health benefits (Radzik-Rant et al., 2018). The effect of a diet supplemented with brewery waste on the growth of fish was investigated as well. BSG was used at the levels of 10–40% to replace rice bran in the diet of catla (*Catla catla*), rohu (*Labeo rohita*), and mrigal (*Cirrhina mrigala*). BSG was shown to exert a positive effect on the absorption and utilization of feed. The body weight gain upon administration of the diet supplemented with 30% BSG was observed in the catla and rohu, but not in the mrigal. Therefore, the studies revealed that BSG addition to the fish diet contributed to better growth performance, although the results depended on the fish species. Improved growth performance was also observed in carps fed on BSG-containing diets, which was associated with the presence of essential amino acids in this waste (Kaur & Saxena, 2004).

Some studies investigated the effects of incorporation of sorghum-barley brewers’ spent grain (SBBSG) into poultry diet. It was shown that up to 16% SBBSG can be safely used in poultry feed since it had no adverse effects on broiler performance and health (it did not influence the blood profile or body weight gain of the broilers). Moreover, it was found to improve the feed conversion efficiency (Nortey, Frimpong & Naazie, 2018).

Despite the advantages of the addition of BSG as animal feed, its use is still limited, which is mainly related to problems with storage of the material. The high moisture content of this residue makes it susceptible to microbial growth in the usual environmental conditions prevailing on farms (Stojceska et al., 2008). Within few days after production, wet grain can undergo molding and spoilage; therefore, preservation is a key factor in the utilization of this by-product. Wet BSG can be preserved by e.g., drying with solar radiation and ensiling (alone or with other dry forages) (Chanie & Fievez, 2017).

Another brewery waste, hot trub, is used as a feed ingredient much less frequently than BGS. Spent hops present in this waste have high fiber content, but also contribute to an unpleasant flavor, which makes hot trub impracticable for direct use in feed. According to some sources, hot trub is not used as animal feed due to the bitterness caused by hops (O’Rourke, 1994). Nonetheless, other feeding experiments with application of products containing a dried trub-yeast mixture have shown that this combination can be used in dried protein feed preparations and, despite the bitterness, the yeast-trub mixture was acceptable as pigs’ feed (RuB & Meyer-Pittroff, 2003).

Spent yeast is used in animal food not only in combination with hot trub. Surplus yeast is often sold to farmers, mostly in a wet form, as an inexpensive animal feed additive. The high digestibility of yeast was confirmed in animals such as rats, pigs, and salmon (Øverland et al., 2013). This residue is a rich source of proteins, vitamins (mainly B group), and minerals (especially phosphorus) that can be used in animal diets (Stone, 2006; Crawshaw, 2004). This brewing by-product was reported as a feed additive for fish, horses, ruminants, poultry, and swine (Ferreira et al., 2010; Tacon, Metian & Hasan, 2009; Crawshaw, 2004; Hertrampf & Piedad-Pascual, 2000). BSY can be used as feed in a fresh, liquid, or dried form. Before administration to animals, yeasts have to be inactivated by heat treatment or by addition of organic acids. It is indispensable to prevent fermentation of this residue after consumption by animals, as it can induce gastro-intestinal problems in pigs (Crawshaw, 2004). Supplements containing brewer’s spent yeast mixed with spent grain were found to positively influence the productive (increased egg production and quality) and reproductive (increased effectiveness of fertilization and hatchability) efficiency inbreeding turkeys and hens. BSY can also replace soya in diets for pigs and sows, enriching the feed with essentials amino acids (Levic, Djuragic & Sredanovic, 2010).

### Food ingredients or additives

It has been evidenced that BSG has a desirable nutritional value for the human diet. The major BSG components, i.e. fiber and proteins, are essential constituents of the human diet and can improve the value of food products. Moreover, the addition of BSG enriches food products with additional beneficial features. It enhance the aroma binding properties and has a positive effect on gelling and emulsifying potential. Since the ingredients used in the brewing process are approved for human consumption, this by-product can be safely applied for the development of new food products, which receive full health-related regulatory approval. Although currently it is not used in large-scale production of food, BSG can be added as a low-cost constituent of food intended for human consumption (Table 1) (Faˇrcaş et al., 2017; Thomas & Rahman, 2006).

**Table 1:** Developed food products utilizing BGS and BSY as ingredients / additives.

**Food products incorporating brewer’s spent grain**	**Reference**
Wheat bread	Plessas et al. (2007)
Brewer’s spent grain bread	Stojceska & Ainsworth, 2008; Ktenioudaki et al. (2015) and Steinmacher et al. (2012)
Spent grain ciabatta	Cina (2015)
Wheat dough supplemented with BSG	Ktenioudaki, O’Shea & Gallagher (2013b)
Breadsticks	Ktenioudaki et al. (2012)
Baked snacks (crispy-slices)	Ktenioudaki et al. (2013a)
Whole grain pizza crust	Combest & Warren (2018)
Whole wheat bagel	Combest & Warren (2018)
Whole wheat muffin	Combest & Warren (2018)
Doughnuts	Lynch, Steffen & Arendt (2016)
Brownies	Lynch, Steffen & Arendt (2016)
Cookies	Kissell, Prentice & Lindsay (1979)
Puffed snacks	ReGrained Company (2020)
Chickpea based snack	Ainsworth et al. (2007)
Ready-to-eat snacks	Stojceska et al. (2008) and Stojceska et al. (2009)
Bars	ReGrained Company (2020)
Waffles	Brooklyn Brew Shop (2017)
Pancakes	Faˇrcaş et al. (2017)
Tortillas	Combest & Warren (2018)
Pasta	Cappa & Alamprese (2017) and Sobukola, Babajide & Ogunsade (2013)
Breakfast cereals	Faˇrcaş et al. (2014)
Germinated barley foodstuff (GBF, protein-rich and fibrous foodstuff)	Kanauchi, Mitsuyama & Araki (2001)
Beef frankfurters	Özvural et al. (2009)
Smoked sausages	Nagy et al. (2017)
Chicken sausages	Choi et al. (2014)
Chicken patties	Kim et al. (2013)
Spent grain chedar scones	Brooklyn Brew Shop (2016)
Vegan Burgers	Rebekah (2013)
**Products with brewer’s spent yeast as food additive**	
Mayonnaise with β- glucan from BSY as a fat replacer	Worrasinchai et al. (2006)
Bread	Martins et al. (2015)
Vegan cake	Coldea et al. (2017)
Cooked ham with 1% BSY extract	Pancrazio et al. (2016)
French salad dressing with mannoprotein from BSY	Melo et al. (2015)
Meat substitutes	Gibson & Dwivedi (1970)
Carrot and beetroot juice with BSY autolysate	Rakin, Baras & Vukasinovic (2004) and Rakin et al. (2007)

Due to the high dietary fiber content, BSG-enriched food may provide some health benefits, such as prevention of certain chronic diseases (coronary heart disease, cancer, diabetes, and gastrointestinal disorders) (Stojceska et al., 2008). The consumption of BSG favorably influences the digestive system, reduces total lipid and cholesterol levels, and can reduce the amount of synthetic antioxidants added to products (Choi et al., 2014; Aliyu & Bala, 2011; Özvural et al., 2009). Addition of BSG enriches bread and pastries with fiber, protein, lipids, and minerals and adds a novel pleasant flavor and good organoleptic attributes (Öztürk et al., 2002). BSG can also be processed into flour. Numerous studies have shown that BSG can be incorporated into flour used for production of many foodstuffs, such as bread, waffles, cookies, pancakes, tortillas, pasta, and breakfast cereals (Waters et al., 2012) (Table 1). BSG is often used to make Spent Grain Bread. Its addition enhances the nutritional value of bread and allows full utilization of the nutritional and taste properties of malt used for beer production. The type of grain used can also be matched to the style of baked bread (for example, the BSG from Pilsner can enrich a light sandwich loaf, while the grain from an imperial stout can be suitable for dark pumpernickel) (Carpenter, 2014). Studies have revealed that BSG addition to wheat flour bread increases the fiber amount and alters the fat content in the product. Incorporation of BSG combined with the use of appropriate enzymes extends the shelf life of bread and improves such properties as texture and loaf volume (Stojceska et al., 2008). Addition of BSG flour to bakery products was shown to result in enhanced water holding capacity and texture of the products and slightly increase their sweetness (Waters et al., 2012; Ktenioudaki, O’Shea & Gallagher, 2013b). BSG was also tested as an ingredient of breadsticks. It significantly increased the dietary fiber and protein content in this product and modified its baking properties by influencing the texture and structure. The breadsticks obtained were darker and less crispy and had a lower baking volume (Ktenioudaki et al., 2013a). Moreover, BSG-containing flour (at the level between 5 and 60%) was used as an ingredient of cookies. Incorporation of 40% of BSG flour contributed to maintenance of appropriate physical quality of the cookies and, simultaneously, significantly enriched this product with N sources and fiber. BSG has also been examined as an ingredient of ready-to-eat snacks and extruded snack food. BSG incorporated into the formulation mix at levels ranging from 10 to 30% had a positive effect on textural and functional properties and, as previously, increased the crude protein and fiber content. Partial replacement of maize flour with BSG (at levels ranging from 10 to 30%) in chickpea snacks increased the protein, fat, and fiber content in the product (Stojceska et al., 2008).

BSG can also be used as a beneficial adjunct to meat products, as it can replace animal protein or/and enrich these products with dietary fiber. There are reports on the utilization of brewer’s spent grain in the production of low-fat beef Frankfurters, smoked sausages, and reduced-fat chicken sausages (Table 1) (Özvural et al., 2009; Nagy et al., 2017; Choi et al., 2014). Dietary fiber extracts from BSG were also used to prepare patties from chicken breast and pork back fat. The studies revealed that 3% of BSG dietary fiber extract could be applied as a source of dietary fiber for enhancing the quality characteristics of meat patties (Kim et al., 2013). Brewer’s spent grain addition has no negative effect on the quality characteristics of produced meat products, but increases the health-promoting properties of food.

Spent yeast can also be applied for production of functional food ingredients (Table 1). This by-product contains many valuable and bioactive substances that are important for human nutrition (Ferreira et al., 2010). The use of this residue is limited by its strong bitter taste resulting from the presence of hops in boiled wort, although there are methods for removal of the taste (Mathias et al., 2017). Given its high mineral content, BSY can bring beneficial effects in other branches of the food industry, including confectionery, dairy industry, and production of beverages, such as juices and mead. BSY has been successfully applied in the bakery industry for production of flour (Rakowska et al., 2017). Addition of BSY to foodstuffs has been found to be beneficial, since it exhibits prebiotic properties (Borchani et al., 2016). BSY is an important source of proteins and essential amino acids. It can be used to formulate new food products and food supplements rich in B-complex vitamins, minerals (selenium, chromium), and polyphenolic compounds with antioxidant activity (Coldea et al., 2017; Podpora et al., 2016). BSY proteins can replace soy proteins as a snack food ingredient, and their higher digestibility is an additional advantage (Ferreira et al., 2010). The by-product was also used for fortification of vegan cake with increased protein, lipid, and carbohydrate content (Coldea et al., 2017). Food products containing BSG and BSY as an additive are summarized in Table 1.

Among brewery wastes, hot trub is the least frequently used in the food industry because of the bitterness originating from its ingredients. However, Saraiva et al. (2019) has developed an extraction process to reduce the bitterness of its taste while maintaining and even improving its characteristics. Hot trub with changed composition and functionability can be used in the food industry for enrichment of fat-rich products or as an alternative source of vegetable protein (Saraiva et al., 2019).

### Extraction of bioactive compounds

Brewery wastes can be used for recovery of some bioactive compounds, which can be further used as functional food ingredients (Faˇrcaş et al., 2017). Some components of BSG, such as arabinoxylans and phenolic compounds, are interesting compounds for application in the food industry due to their properties and potential health benefits (Severini et al., 2015).

BSG residues are rich in phenolic acids, especially ferulic and p-coumaric acids, which are contained in the husk and cell wall of grain used for the brewing process and remain in this by-product (Stefanello et al., 2018; Bartolomé, Faulds & Williamson, 1997). Hence, BSG can potentially serve as an inexpensive source for extraction of these valuable compounds. Many studies have revealed that phenolic acids can be extracted from BSG. The most commonly applied methods consist in liquid-liquid or liquid-solid extraction (with such solvents as methanol and ethyl acetate), acid hydrolysis, and saponification (with NaOH) (Stalikas, 2007; McCarthy et al., 2013). Some researchers have tested the efficiency of novel extraction techniques for this purpose, such as the rapid microwave-assisted derivatization process (Athanasios et al., 2007).

Ferulic acid (4-hydroxy-3-methoxy-cinnamic acid) is a phenolic acid with a variety of potential applications as a natural antioxidant, photoprotectant, food flavor precursor, and food preservative/antimicrobial and anti-inflammatory agent. p-Coumaric acid (4-hydroxycinnamic acid) can serve as a chemoprotectant and antioxidant (Bartolomé et al., 2002; Faulds, Sancho & Bartolomé, 2002). Ferulic acid was successfully extracted from BSG via alkaline hydrolysis, with a yield of 0.3% (Bartolomé, Faulds & Williamson, 1997; Aliyu & Bala, 2011). Studies have revealed that the amount of ferulic acid released from BSG can be increased (3.3%) by application of esterase from *Aspergillus niger* or xylanase and esterase secreted by* Trichoderma viride* growing on BSG. An increase in the content of ferulic acid obtained from BCG was also observed when crude *Fusarium oxysporum* was used (Xiros et al., 2009). It has also been reported that β-glucanase from *Humicola insolens* can effectively release available ferulic acid from BSG (Faulds, Sancho & Bartolomé, 2002).

It has been shown that polyphenols and flavonoids can be extracted from BSG using supercritical CO_2_.This method coupled with further microencapsulation of these compounds masks their unwanted bitter taste in food products fortified with these bioactive ingredients. Moreover, this method preserves the stability of polyphenols derived from BSG. Microencapsulation not only prevents degradation of bioactive compounds derived from BSG, but is also used to minimize the unpleasant sensorial properties and appearance of this by-product. Production of BSG extracts and microencapsulation make it more attractive for use in human food products while maintaining its desirable properties. Microencapsulated polyphenols obtained with this method were applied as an additive to fish burgers. The product was richer in phenolic compounds and flavonoids than the control sample and exhibited higher antioxidant activity, which makes BSG an attractive food supplement (Spinelli, Conte & Del Nobile, 2016).

BSG has also been used for extraction of arabinoxylans and proteins in an integrated process of sequential extraction of proteins and arabinoxylans from BSG with increasing concentrations of alkali (KOH or NaOH). This extraction process is characterized by good efficiency, i.e., 82–85% of total proteins and 66–73% of total AX (Vieira et al., 2014), and the products obtained can be subsequently used as functional food ingredients (Rosicka et al., 2015). With its properties, arabinoxylan can be used as a film-forming and surface active agent or cryostabilizer in food products. It is used in the food industry, as it can influence the water-holding capacity of food and dough starch retrogradation and can improve the quality and properties of bread (Cui, Wu & Ding, 2013). Moreover, BSG has been tested as a potential raw material for production of cellulose nanofibres, which are applied in the food industry, for example as emulsion/dispersion agents (Klemm et al., 2011).

Since it is rich in proteins (74–78% of malt protein remains insoluble in the BSG), BSG can be potentially used as a healthy functional food, similarly to whey protein. Essential amino acids constitute approximately 30% of the total BSG protein content (Faˇrcaş et al., 2017). BSG usually contains large amounts of lysine, leucine, phenylalanine, isoleucine, threonine, and tryptophan, although the amino acid profiles of BSG may vary significantly, depending on the type of malt used in the brewing process (Waters et al., 2012). However, BSG proteins are not widely used in food products due to their insolubility (Faˇrcaş et al., 2017). It has been shown that the insoluble protein fraction obtained from BSG can be used as a substrate for production of hydrolysates, which can be incorporated into food products (Celus, Brijs & Delcour, 2007; Vieira et al., 2014). Protein hydrolysates obtained from BSG exhibit various biological properties desirable in the food industry, e.g. emulsifying, antimicrobial, anti-inflammatory, and immunomodulatory activity (Crowley et al., 2015; McCarthy et al., 2013).

Spent hops are another by-product of the brewing industry used for extraction of useful compounds. It has been reported that this waste can be a source of compounds used for protection of stored food. Essential oils from spent hops were characterized after extraction using hydrodistillation. Their yield reached 0.11% of dry weight of the material. The main essential oils obtained from this by-product (myrcene, *α*-humulene, and β-caryophyllene) exhibit repellent activity and represent an eco-friendly and low-cost alternative to synthetic insect pest repellents used for protection of stored food-stuff (Bedini et al., 2015).

Due to its high moisture content and chemical composition, post-fermentation yeast biomass is vulnerable to rapid degradation, which makes the storage of BSY without previous preservation difficult. However, BSY can be successfully used as a source of food-grade yeast extracts (Ravishankar, 2016). Furthermore, hydrolyzed BSY can potentially yield a raw material for use in the food industry. This substrate derived from BSY has a potential to be applied in the wine industry as a fermentation-activating compound. It is also possible to use this raw material as a source of bioactive peptides. These health-stimulating compounds introduced into food support the human organism in removal of free radicals due to their anti-oxidant properties (Podpora et al., 2016).

The production of autolysates from BSY is attractive to manufacturers, since it brings high profits from the use of inexpensive raw materials for the production of value-added compounds. A process of cell lysis induced by saponin action, which is a simple and low-cost procedure, has been developed. It provides pro-health ingredients for functional foods and beverages (Berlowska et al., 2017). Yeast extracts obtained from BSY by autolysis vary in the free amino acids content (depending on the strain used) and can contain peptides with different molecular weights. For this reason, these various yeast extracts can be adapted to specific nutritional needs as an additive for functional foods and dietary supplements. These extracts leave a bitter aftertaste, which is mainly a result of the high content of free glutamic acid released from proteins during hydrolysis. This can be an advantage during creation of new functional foods with this particular taste profile (Podpora et al., 2016). Yeast extracts obtained from BSY may serve as natural flavor enhancers. A combination of compounds contained in the extracts, such as 5-nucleotides, peptides, and amino acids (especially glutamic acid), is responsible for improving the flavor of food products and spice mixtures. This safe ingredient can potentially replace glutamates and protein hydrolysates that are commonly added to processed foods (Ferreira et al., 2010; Podpora et al., 2016). Yeast extract can also serve as a source of peptides and free amino acids included in functional foods. However, the amount of yeast extract obtained from BSY that can be used in food is limited by its sensory quality (Coldea et al., 2017).

Yeast extracts from BSY have been found to be strong antioxidants, since BSY exhibits high antioxidant activity (22.18–32.73 mMol TEAC/100 ml) due to the presence of polyphenolic compounds (Podpora et al., 2016). These substances are absorbed by yeasts from the external medium, which in the case of beer brewing is rich in phenolic and polyphenolic compounds derived from BSG and hot trub (Vieira et al., 2016). Substances with antioxidant activity are used in food, since they can prevent or delay some types of cell damage caused by the oxidation of biologically relevant molecules (Shahidi & Ambigaipalan, 2015). Inclusion of antioxidants into diet can lower the risk of development of certain diseases (cancer, cardiovascular and neurodegenerative diseases) (Huang, 2018).

The use of enzymatic hydrolysis and autolysis processes enables to obtain from BSY fractions that can be subjected to selective membrane filtration. It allows to recover four fractions which exhibit different molecular weights (with protein and sugar contents between 30-69% and 20-48%, respectively). This process yields nutritional ingredients extracted from BSY (protein, minerals, and carbohydrates), which can be incorporated into dietary products. These compounds are also useful for the food industry because of their mineral content (they are rich in sodium and potassium) and amino acids content (high level of glutamine, glutamic acid, and alanine) (Amorim et al., 2016).

BSY contains compounds that are valuable for the food industry, e.g. nucleic acids and vitamin D, which can be extracted from yeast (Tacon, 2015; Hertrampf & Piedad-Pascual, 2000). BSY is an inexpensive source of a raw material used for the enrichment with D_2_ (ergocalciferol) from the precursor ergosterol. Vitamin D _2_ derived from yeasts is a source of vitamin D, which can be applied as a dietary supplement in vegan food products (Metzger, Scholl & Barnes, 2012). Since BSY contains approximately 10% of nucleic acids, it is an excellent source for large-scale production of the aforementioned 5’-nucleotides. They are used in the food industry as taste and scent enhancers e.g. in soups, bouillons, and gravies. They are used in small amounts and can replace beef extract, which is currently widely applied as a flavor enhancer (El-Aleem et al., 2017).

Brewer’s spent yeasts are an important source of some valuable saccharides with a wide variety of molecular weights, such as β-glucans or mono-, di-, and oligosaccharides (e.g. trehalose and mannans). Glucan is able to bind water and has therefore been successfully used in the food industry as a water retention additive, thickening agent, etc (Krpan et al., 2009). In recent years, β -glucans have drawn researchers’ attention due to their pro-health properties (Podpora et al., 2016; Rakowska et al., 2017). β-glucan obtained from BSY has been reported to exhibit multi-directional biological activity. This compound improves the immunological system of humans and other animals, exhibits prebiotic and antioxidant activity, and positively influences blood lipid content (Rakowska et al., 2017; Otero et al., 2011). These properties make glucans attractive supplements for functional food, and BSY is a good source for extraction of this compound (Waszkiewicz-Robak, Karwowska & Swiderski, 2005).

Another potential application of BSY can be the production of trehalose, which is synthesized by yeasts during fermentation (Mahmud, Hirasawa & Shimizu, 2010; Benaroudj, Lee & Goldberg, 2001; Rúa et al., 2008). It has been reported that it can be extracted from BSY using high-intensity pulsed electric fields (PEF) (efficiency 103.15 g/s). Trehalose is a highly stable disaccharide composed of two glucose molecules. It is widely used in the food industry as a food additive (Jin et al., 2011). Trehalose is known to be a good bio-protectant of biomolecules against freezing. This property makes it an excellent supplement for foods that undergo the freezing and drying process (Otero et al., 2011). It is also used to improve the texture of food, release food flavor, and stabilize proteins contained in food. It is less sweet than sucrose (45% of its sweetness). Due to its humectant activity, trehalose is added to a wide variety of products, such as confectionery, bread, ice creams, and soft drinks. Crude extracts from BSY have also been found to be a source of invertase, i.e., an enzyme converting sucrose and polysaccharides to fructose and glucose (De León-González et al., 2016). Invertases are used in the food industry, mainly in confectionery, as a catalytic agent in production of artificial sweeteners (Veana et al., 2018).

### Application of brewery wastes in other branches of the food industry

BSG derived from grain is rich in arabinoxylan and ligninocellulose, i.e. materials with high content of polysaccharides. These compounds can be subjected to hydrolysis (enzymatic, acidic, or hydrothermal) and degraded into their constituents. As a result of hydrolysis, glucose can be obtained from cellulose, whereas hemicellulose is degraded into arabinose, xylose, mannose, galactose, acetic acid, and hydroxycinnamic acids (Wyman et al., 2005). These products can be further used as a substrate for the fermentation process and yield other valuable compounds. Due to its high polysaccharide, protein, nutrient, and water content, BSG is susceptible to degradation by microorganisms and promotes their growth (Mathias et al., 2015). Therefore, there are attempts to use this residue in biotechnological processes. BSG biomass can be exploited via fungal and bacterial processing to obtain enzymes and value-added compounds for the food industry (Ravindran & Jaiswal, 2016). Similarly, hydrolysates produced from BSG can be subjected to fermentation processes to produce various compounds for the food industry. The valuable chemicals obtained can serve as raw materials for further processing or become functional ingredients for production of new functional food products (Lynch, Steffen & Arendt, 2016). BSG can be utilized as a growth substrate for microorganisms that are able to degrade the fibrous husk materials contained in this by-product. It has been shown that media formulated with BSG contain nutrients that support the growth of such bacteria as *Escherichia coli*, (Archibong et al., 2016), actinobacteria (Szponar et al., 2003), *Bifidobacterium adolescentis* 94BIM, and *Lactobacillus spp.* (Novik et al., 2007)*.* Actinobacteria and *E. coli* are used for the production of a wide range of bioactive metabolites, including enzymes and metabolites applied in the food industry (Ramírez & Calzadíaz, 2016; Kallscheuer, 2018). In turn, *Bifidobacterium* and *Lactobacillus* are most widely used probiotics in food products (Song, Hayek & Ibrahim, 2012). Moreover, BSG has been effectively used for cultivation of fungi, among others *Pleurotus ostreatus* (Gregori et al., 2008), *Penicillium brasilianum* (Panagiotou, Granouillet & Olsson, 2006) and *Rhodosporidium toruloides* (Cooray, Lee & Chen, 2017). BSG was also successfully used as an alternative to expensive nitrogen sources for preparation of media for yeast cultivation. Growth media containing fermented BSG were tested on the *Rhodosporidium toruloides*, and the by-product was able to support the growth of this yeast at comparable levels as YPD, sustaining its normal metabolic activity. *Rhodosporidium toruloides* cultivated on BSG-based media produced fatty acids and carotenoids, which can be used in food products, for example as natural colorants (Cooray, Lee & Chen, 2017; Zalynthios & Varzakas, 2016). Brewery spent grain can substitute yeast extract-peptone, which is usually used for cultivation of fungi producing useful and valuable bio products, such as succinic acid (Cooray, Lee & Chen, 2017), microbial oil (Saenge et al., 2011), xylitol (Mussatto & Roberto, 2008), or pullulan (Singh & Saini, 2012; Mussatto & Roberto, 2008; Cooray, Lee & Chen, 2017). Processes for production of xylitol from BSG have been developed. Xylitol is a sugar occurring in nature in small amounts. In the food industry, it is used as a sweetener and a healthier alternative to sucrose. It is beneficial for prevention of lung infection and dental caries and recommended for diabetics and patients suffering from renal lesions and lipid metabolism disorders (Mussatto & Roberto, 2008). It has been indicated that such yeasts as *Debaryomyces hansenii* (Carvalheiro et al., 2006) and *Candida guilliermondii* growing on BSG produce xylitol (Mussatto & Roberto, 2008). Another metabolite that can be produced on BSG is an extracellular water-soluble polysaccharide named pullulan. It is a linear *α*-D-glucan produced by the fungus *Aureobasidium pullulans,* consisting mainly of maltotriose units with α-(1 →6) or (1 →4) linkages. Fermentation of medium based on BSG by *A. pullulans* yields 6.0 g/l of pullulan after 72 h linkages (Roukas, 1998). Dietary pullulan functions as a prebiotic promoting the growth of beneficial bifidobacteria (Sugawa-Katayama et al., 1994). This polysaccharide can replace starch in some foods; it can also be used as a binder and stabilizer in food paste or as low-viscosity filler in beverages and sauces. Pullulan can be incorporated into dietetic foods beneficial for diabetics or patients with impaired glucose tolerance (Singh & Saini, 2012).

Brewer’s spent grain has been found to be a less expensive alternative to the traditional carbon sources (such as glucose, sucrose, or starch) for production of L-lactic acid (2-hydroxy propanoic acid). This compound can be found in milk, dairy products, and many other fermented food products (pickled vegetables, jams, frozen desserts). This natural food additive is used e.g. as a preservative, pH regulator, flavor, solvent, and gelling agent (Ali, Anjum & Zahoor, 2009; Ameen & Caruso, 2017). Studies have demonstrated that BSG hydrolysate can be used as a substrate for lactic acid production by *Lactobacillus delbrueckii, Lactobacillus pentosus,* or *Lactobacillus rhamnosus* NBRC14710 (Cruz et al., 2007; Shindo & Tachibana, 2004).

BSG has also been tested as an inexpensive substrate for citric acid production. Citric acid is a widely used compound serving as an antioxidant, flavor enhancer, and preservation agent in food and beverages. Considerable amounts of citric acid were produced from BSG-utilizing *Aspergillus niger* and *Saccharomyces cerevisiae* in submerged fermentation (0.512% and 0.312%, respectively) (Femi-ola & Atere, 2013). Hydrolysates obtained from BSG have also been successfully applied as a fermentation medium for production of ethanol by *Saccharomyces cerevisiae* and* Debaryomyces hansenii* or glycerol and arabitol by *Debaryomyces hansenii* (Laws & Waites, 1986; Duarte et al., 2004; Carvalheiro et al., 2006).

Microorganisms growing on BSG produce various types of enzymes that can be used in food processing. For this reason, BSG can also be applied as a low-cost and widely available substrate for enzyme production by bacteria. The type and activity of produced enzymes depends on the cultivated strain and exact growth substrate composition. BSG is a potential substrate for cultivation of amylolytic organisms, which are known to produce β-amylase and amyloglucosidase (Adeniran & Abiose, 2009). Such microorganisms as *Aspergillus oryzae* and *Bacillus* have been used for production of α-amylase (Xu et al., 2008; Hashemi et al., 2011). In the food industry, amylases are used for production of fructose and maltotetraose syrup, oligosaccharide mixtures, or for reduction of starch viscosity during liquefaction (Adeniran & Abiose, 2009). BSG has been demonstrated to promote the production of cellulases, hemicellulases, endoxylanases, β-xylosidases, α- arabinofuranosidases, and feruloyl esterases used by microorganisms to degrade hemicellulose contained in this residue (Mandalari et al., 2008; Panagiotou, Granouillet & Olsson, 2006). These enzymes have been applied in food processing. There are some examples indicating the use of *Aspergillus fumigatus, Fusarium oxysporum*, and *Streptomyces malaysiensis* for production of cellulases (Grigorevski-Lima et al., 2009; Xiros et al., 2009; Nascimento et al., 2009). BSG was also applied as a carbon source for production of xylanolytic enzymes by *Penicillium janczewskii* and feruloyl esterase by *Talaromyces stipitatus, Humicola grisea var. thermoidea* (Mandalari et al., 2008), and *Streptomyces avermitilis* CECT 3339. The latter strain growing on BGS was also found to produce (1 → 4)- β-D-xylan xilanohydrolase (Bartolomé et al., 2003). Xylanolytic enzymes are widely used in the food industry. With their properties, they can be applied in bread and pastry to improve their quality or for removal of coffee bean mucilage. These enzymes are also used to improve the digestibility of crops intended for ruminant feed (Bajpai, 2014).

Since the carbon/nitrogen ratio in hot trub is similar to that in the microbial cell composition, it is suggested that, like BSG, this residue can be applied as an additive in fermentation media used in bioprocesses, as it exerts a positive effect on cell divisions (Mathias et al., 2015). Potentially, it can be effectively used as a supplement in cultures of some microbes producing compounds for the food industry.

BSY can be used as an additive to media for cultivation of microorganisms producing relevant value-added compounds used in the food industry. For this purpose, mainly hydrolysates and autolysates obtained from BSY are used. BSY is a potential supplement to media utilized for the growth of lactic acid bacteria and production of lactic acid, for production of ethanol by genetically modified *E. coli* strain, and for synthesis of succinic acid. The applications of BSY as a source of nutrients in microbiological media have been summarized by Ferreira et al. (2010). Microorganisms growing on BSY release extracellular proteolytic enzymes. This residue has been reported to have the highest potential to be used as an additive to media for producing proteases. BSY has also been shown to be a promising alternative nitrogen source for microbial growth (Mathias et al., 2017). With their chemical properties, also yeast autolysates obtained from BSY can be used as supplements for fermentation media. They are suitable for the growth of microorganisms producing specific products that can be used in special-purpose foods (Berlowska et al., 2017).

## Conclusions

Beer is one of the most frequently consumed beverages worldwide. Yet, a huge amount of waste is generated during beer production. Approximately 85% of by-products generated in this process can be changed into valuable resources, thus significantly reducing production costs and at the same time contributing to an increase in self-sufficiency. The by-products reviewed in this article have a potential to be used in products that are vital for human and animal nutrition and to extract some compounds for the food industry and culture of microorganisms in industrial bioprocesses, which yield valuable compounds for the food industry. However, disposal of wastes in an environmentally sustainable manner is an important challenge. There is still a need to design and develop feasible and financially viable processes for utilization or revalorization of brewery waste. Efficient management of these by-products can be ensured by a solution aimed at limiting the environmental pollution hazard caused by the disposal thereof, simultaneously making them useful for the food industry. For better environmental and economic performance, these by-products should be converted into some valuable products instead of being considered as useless wastes. Utilization thereof will reduce the costs of food and feed production and will allow taking full advantage of the nutritional value of this waste. While some attempts have been made to incorporate the bioactive components of BSG and BSY into foodstuffs, further research in this area should be conducted.

## Additional Information and Declarations

### Data Deposition

The following information was supplied regarding data availability: This is a review article. There is no code included and no data from research experiments.
